# Supplementary material for: Mechanical Properties Optimization of Hybrid Aramid and Jute Fabrics-Reinforced Graphene Nanoplatelets in Functionalized HDPE Matrix Nanocomposites
Source: Polymers (Basel). 2023 May 26;15(11):2460. doi: 10.3390/polym15112460 (PMC10255711; doi:10.3390/polym15112460)
Supplement: Supplementary file 1 [file polymers-15-02460-s001.zip › polymers-2403515-supplementary/Supplementary tables.pdf]

Table S1. Raman shift values of the Raman spectrum of neat HDPE.

Index	Position (cm^{-1})	Intensity (FIT) (u.a.)	FWHM
I	1060.8	46800	21.2
II	1127.7	54900	20
III	1168	10000	15.6
IV	1294.2	72300	20
V	1367.3	3500	16.8
VI	1418.7	30500	15.6
VII	1439.8	62900	18
VIII	1459.4	45300	25.8
IX	2721.1	41900	32
X	2849.7	539500	27.4
XI	2883.8	649800	24
XII	2907.7	152200	28
XIII	2930.9	117500	28
XIV	2998	13200	32.2
XV	3031.5	13200	29.8
XVI	3068.5	4800	26.4

Table S2. Raman shift values of the Raman spectrum of GNP/HDPE nanocomposite.

Index	Position (cm^{-1})	Intensity (FIT) (u.a.)	FWHM
I	1062	40500	20
II	1128	46800	16
III	1166.8	7200	15.8
IV	1295.1	62000	17.2
V	1367.5	3200	14
VI	1416.21	22550	16
VII	1440.11	61250	22
VIII	1461.57	34080	24
IX	1584.9	4800	29
X	2722.7	43600	32
XI	2850.46	443800	26
XII	2883.4	534900	24
XIII	2906.4	126300	24
XIV	2928.24	115700	30
XV	2993.9	9400	60
XVI	3030.2	12700	44.2
XVII	3071.3	4600	33.6

Table S3. Raman shift values of the Raman spectrum of GNP.

Index	Position (cm^{-1})	Intensity (FIT) (u.a.)	FWHM
I	1347.7	1240	47.6
II	1573.6	12350	24
III	2715.9	4168	76
