# Supplementary figures and images for: Mechanical Properties Optimization of Hybrid Aramid and Jute Fabrics-Reinforced Graphene Nanoplatelets in Functionalized HDPE Matrix Nanocomposites
Source: Polymers (Basel). 2023 May 26;15(11):2460. doi: 10.3390/polym15112460 (PMC10255711; doi:10.3390/polym15112460)

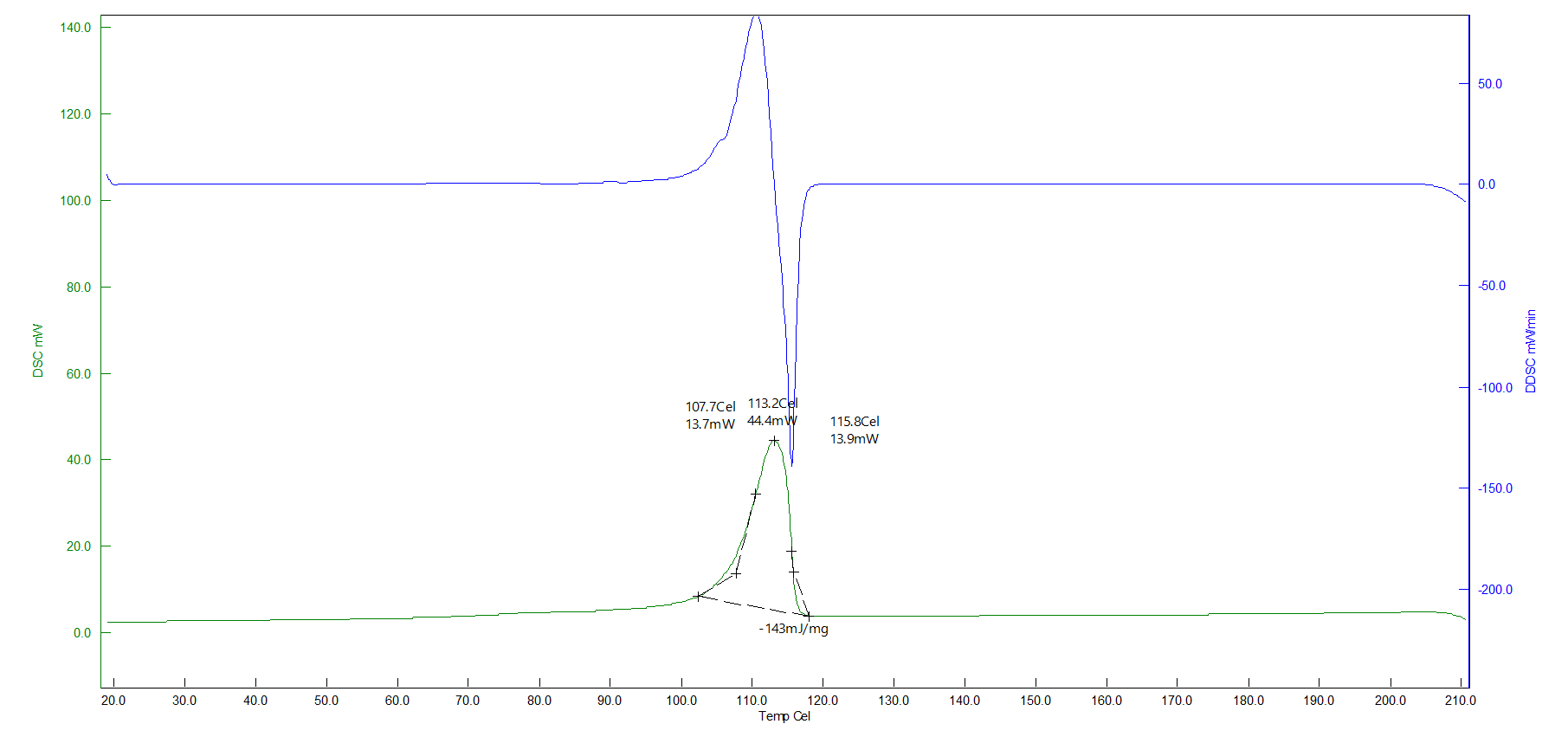

Supplement: Supplementary file 1 [file polymers-15-02460-s001.zip › polymers-2403515-supplementary/Figure S1.tif]

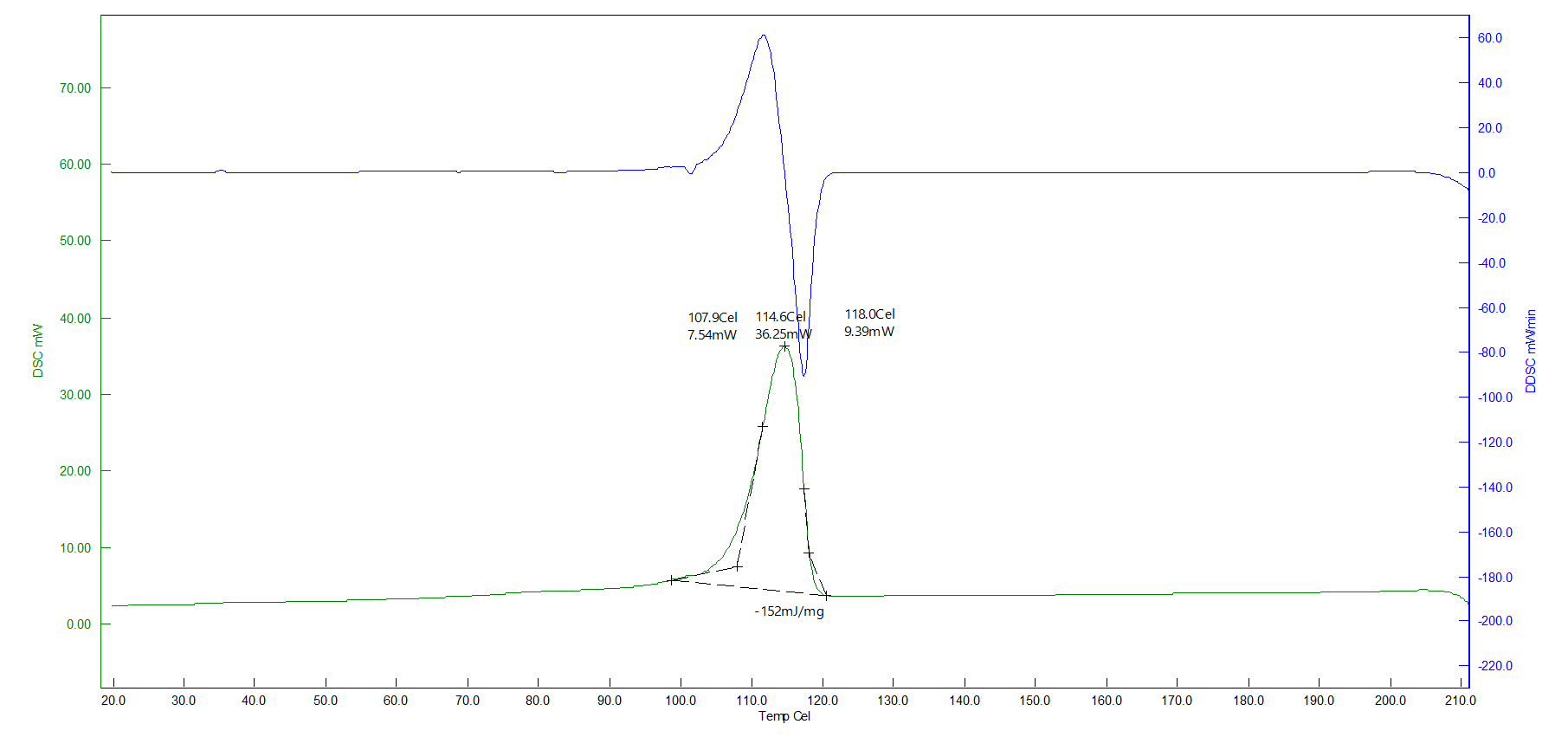

Supplement: Supplementary file 1 [file polymers-15-02460-s001.zip › polymers-2403515-supplementary/Figure S2.tif]

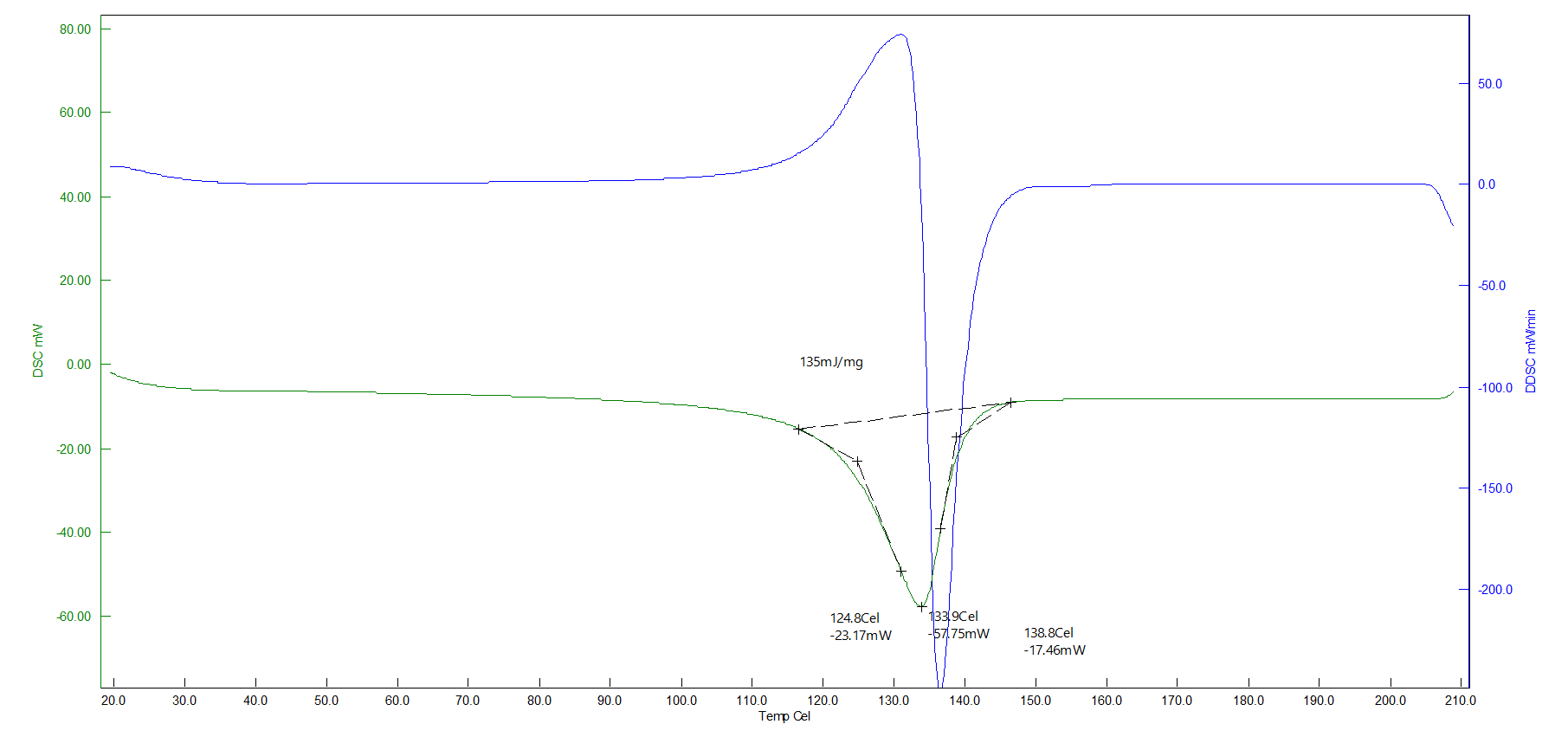

Supplement: Supplementary file 1 [file polymers-15-02460-s001.zip › polymers-2403515-supplementary/Figure S3.tif]

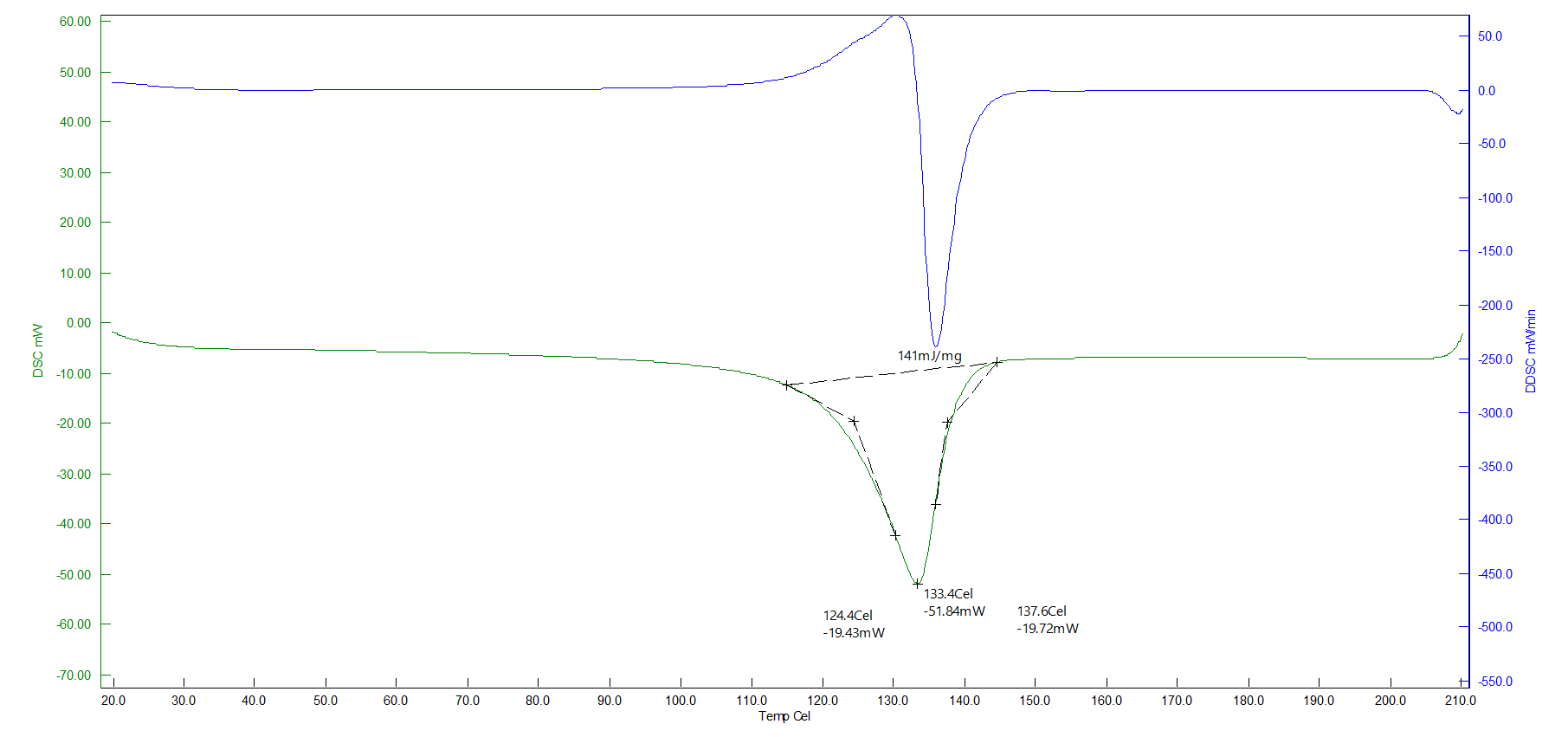

Supplement: Supplementary file 1 [file polymers-15-02460-s001.zip › polymers-2403515-supplementary/Figure S4.tif]
